# Creolin

**Published:** 1891-07

**Authors:** Wm. H. Potter


ARTICLE III.

### CREOLIN.

BY DR. WM. H. POTTER.

[Read before the American Academy of Dental Science, Boston, October 8, 1890.]

All antiseptic drugs are of interest to the dentist. Though there are several antiseptics whose reliable qualities have been thoroughly proven, yet each one possesses one or more serious defects. Hence it is that we look forward to each new antiseptic with the hope that it may possess all the advantages of the old drugs aud none of their disadvantages. The claim has been made that creolin is an improvement over carbolic acid, corrosive sublimate, or iodoform, which constitute our most important antiseptic drugs. In order to judge, if possible, of the value of this claim, I have undertaken to study the literature of the subject, and examine evidence for and against this new preparation.

One and one-half creolin, according to the analysis of Professor H B. Hill, of the Howard Medical School, is a mixture of the sodium salts of some resinous acids with the part of oil of tar known as "iron oil." He thinks the sodium salts are probably what is known as "Harzeife," a resin soap. The oil contains a small amount of carbolic or cresylic acid according to Dr. Hill. Other observers report the absence of any trace of carbolic acid. Pearson & Co., Hamburg, who offer it in the market, give the following analysis:

| | Per cent. |
|---|---|
| Neutral hydrocarbonates, - | 66. |
| Phenols (without carbolic acid) - | 27.4 |
| Organic bases - - - | 2.2 |
| Ash, - - - - | 4.4 |
| | 100.00 |

Creolin is made from English pit-coal by distillation, and is of brownish-black color, and syrupy consistency. It is not a definite chemical compound, but a mixture of various ingredients. It is insoluble in water, but quickly forms with it a brownish or milky emulsion. It also mixes with oil and glycerine. In absolute and ninety-five per cent. alcohol it is soluble in all proportions; also in chloroform and ether. It is non-volatile.

Creolin was mainly used in veterinary medicine previous to the year 1887. In that year its properties were investigated by Esmarch and Eisenberg, and by them the drug was brought to the notice of general practitioners.

According to Dr. Jessner, * the bacteriological works

* *Therapeutic Gazette*, 1889, p. 830.

of Esmarch and Eisenberg show that a three-per-cent. solution of creolin equals a five-per-cent. solution of carbolic acid in antiseptic value.

The same observers † claim that a one-per-cent. solution of creolin retards the development of bacteria better than an equal strength of carbolic acid, and that a five-per-cent. solution of creolin destroys pathogenic bacteria.

Dr. Spaeth ‡ and others took daily doses of eight grammes of creolin for sometime without experiencing any bad result. There was noticed decrease in intestinal gas, inodorous fæces, urine which remained a long time without undergoing ammoniacal fermentation. A patient of Dr. Korturn by mistake took sixty grammes of creolin in five-per-cent. solution without bad result.

Esmarch experimented wtth decomposing material, the germs of Asiatic cholera, typhoid fever, anthrax. Creolin proved a more powerful germicide than carbolic acid, except in the case of the bacilli and spores of anthrax.

At the beginning of the present year, the German opinions upon creolin are summed up in *Braithwaite's Retrospect* * as follows:

Dr. P. Baumgarten states that he has found it to be a strong animal poison and also an antiseptic, yet only in solutions of a strength that would be fatal to animal life.

Dr. Behring comes to the conclusion that creolin possesses antiseptic properties three or four times as weak as those of carbolic acid in solutions of equal strength. A two-per-cent. solution of creolin will not disinfect wounds. The drug is not so poisonous as carbolic acid.

Dr. Eisenberg states that he has found two-per-cent. to five-per-cent solutions of creolin to be powerfully antiseptic, killing almost immediately streptococci, staphylococci, cholera bacilli and other bacteria. Seidel and Hornicke agree with Eisenberg.

---

† Ibid.

‡ *Medical News*, December 1, 1888.

* January, 1890, p. 243.

Hunermann, as the result of a long series of investigations, states that creolin has no right among antiseptics. It undoubtedly retards and in some cases even prevents the further growth of bacteria, but does not kill them. Dr. Korturn and Dr. Bunsen have tried the drug in obstetrical practice, and say that they have found it a most admirable antiseptic, not being so irritating as carbolic acid, and possessing styptic properties. For washing the vagina, Bunsen uses a one-half-per-cent. solution. Dr. Rausche highly lauds the deodorizing properties of creolin, and uses it as an application for burns, superficial wounds, etc.; he also advocates its use in form of soap for cleansing and deodorizing the hands. Drs. Amon and Prutscher have used a weak solution of creolin in aptic and aural surgery to great advantage. From the references quoted it would seem pretty well established, in spite of some contradictory evidence, that creolin possesses decided germicidal power, and that in quantities liable to be used it is non-toxic. It has also proved to be a reliable hæmostatic and a powerful deodorant. It does not injure the hands or instruments. Having such qualities, it has come to be used advantageously in the ordinary routine of antiseptic surgery for disinfecting instruments and hands and for irrigating wounds; also in the treatment of inflammation of the ear and eye, in catarrhal conditions of the nose and pharynx, in cystitis, gonorrhœa, vaginitis, endometritis, and in the antiseptic treatment of labor. It is also efficacious in removing the odor of cancer and in the treatment of burns. Granting that creolin is an efficient germicide, it possesses advantages over carbolic acid. It does not roughen the hands, as does carbolic. The full strength of the drug can be put on the skin with impunity. It is practically non-poisonous, while carbolic is a violent poison, needing to be carefully guarded. It is more agreeable to mucous surfaces than is carbolic, tending to prevent excessive secretion and to reduce congestion. When compared with corrosive sublimate, it can be said to have the same advantages over it that it possesses over carbolic—namely, in being non-toxic, non-irritating, non-depressing as regards mucous surfaces.

Dr. E. O. Otis* makes the statement that creolin combines the favorable workings of iodoform and corrosive sublimate.

The drug has, however, certain disadvantages, not the least of which is the fact that it is a secret preparation. Pearson & Co., of Hamburg, are the most reliable manufacturers, and they guarantee to keep the article up to standard strength and quality. And it has been proved by laboratory experiments that the virtue of the drug has been quite constantly maintained. The negative experiments, however, of certain observers might be explained by supposing some specimens of creolin to be less powerful than others. The opacity of creolin is a disadvantage, since it obscures instruments immersed in it and hides surfaces under treatment. When an emulsion is allowed to stand, a gummy, resinous mass is precipitated, and this may adhere to instruments left in the fluid, making them sticky and difficult to clean. The faults of creolin are, however, not serious, and it is not surprising that the drug has entered into our dental materia medica. It is useful in several ways. First, as a mouth-wash. For this purpose one or two drops of creolin are added to half a tumblerful of water. The emulsion thus made is practically inexpensive. Four or five ounces of the drug, which is quite cheap, will furnish a mouth wash three times a day for a year. Being a powerful deodorant, it corrects disagreeable odors connected with many well-known conditions of the teeth and mouth. Being a reliable germicide, it will at least hold in check the growth of acid-producing germs. Being non-poisonous, it can be prescribed freely and placed in the hands of patients without fear of untoward results. Its slightly astringent action upon mucous

---

* *Boston Medical and Surgical Journal*, August, 9, 1888.

surfaces gives it a peculiar range of usefulness in soft and congested mouths. Its slightly alkaline reaction fits it to correct acid conditions, and, finally, and of no small consequence, its taste of a tarry nature is agreeable to most people. A second sphere of usefulness is in the treatment of fistulous tracts or any suppurating surface. In such cases it tends to diminish secretion and induce a healthy state of the tissues. It is also a good drug for root-canals. It will quickly deodorize a dead pulp, and it makes a reliable antiseptic for the subsequent treatment. If desirable, it can be mixed with alcohol in any proportion, and thus an antiseptic and drying action can be at once secured. In short, it can be used wherever we would use carbolic acid, except in cases where we wish to produce a cauterizing effect or relieve pain.

A minor use to which creolin may be put is that of taking rust off of instruments. To accomplish this the full strength should be used, and the instrument rubbed with a rough cloth or a piece of Faber's rubber and corundum ink eraser. A convenient way of using it is to apply to a felt laboratory wheel.

As to the strength of creolin solutions, a two-per cent. is commonly used for instruments and hands, and a one-half to one-per-cent. for irrigation of wounds and treatment of mucous surfaces.

If any wish to read up on creolin, I would refer them to a very complete and carefully-written article by Dr. E. O. Otis, of Boston, printed in the *Boston Medical and Surgical Journal*, June 20, 1889.

In conclusion, let me say that though creolin promises to be a valuable drug, yet it is still quite new, and we must experiment carefully with it till its true place of usefulness is established. Meanwhile, we should not entirely abandon carbolic acid, corrosive sublimate, or any of the old drugs which have proved themselves efficient in our hands.—*International Dental Journal.*

